# Acupuncture and dry needling for physical therapy of scar: a systematic review

**DOI:** 10.1186/s12906-023-04301-4

**Published:** 2024-01-02

**Authors:** Daria Chmielewska, Jitka Malá, Agnieszka Opala-Berdzik, Magdalena Nocuń, Patrycja Dolibog, Paweł T. Dolibog, Magdalena Stania, Michał Kuszewski, Alena Kobesova

**Affiliations:** 1 Electromyography and Pelvic Floor Muscles Laboratory, Department of Physical Medicine, Institute of Physiotherapy and Health Sciences, Academy of Physical Education in Katowice, Mikołowska 72 street, Katowice, 40-065 Poland; 2 Physiotherapy Department, Faculty of Physical Education and Sport, Charles University in Prague, José Martího 31, Prague 6, 162 52 Czech Republic; 3 Institute of Physiotherapy and Health Sciences, Department of Physiotherapy in Internal Diseases, Academy of Physical Education in Katowice, Mikołowska 72 street, Katowice, 40-065 Poland; 4 Students Scientific Association “IMPULSE” of the Institute of Electromyography and Pelvic Floor Muscles Laboratory, Department of Physical Medicine, Physiotherapy and Health Sciences, Academy of Physical Education in Katowice, Mikołowska 72 street, Katowice, 40-065 Poland; 5 Department of Medical Biophysics, Faculty of Medical Sciences, Medical University of Silesia, Medyków 18 street, 40-752, Katowice, Poland; 6 Department of Biophysics, Faculty of Medical Sciences in Zabrze, Medical University of Silesia, Jordana 19 street, Zabrze, 41-808 Poland; 7 Institute of Sport Sciences, Academy of Physical Education in Katowice, Mikołowska 72 street, Katowice, 40-065 Poland; 8 Institute of Physiotherapy and Health Sciences, Academy of Physical Education in Katowice, Mikołowska 72 street, Katowice, 40-065 Poland; 9 Department of Rehabilitation and Sports Medicine, 2nd Faculty of Medicine, Charles University and University Hospital Motol in Prague, V Úvalu 84, Prague 5, 150 06 Czech Republic

**Keywords:** Dry needling, Acupuncture, Scar, Treatment

## Abstract

**Background:**

There is a continuing interest in finding effective methods for scar treatment. Dry needling is gaining popularity in physiotherapy and is defined by Western medicine as a type of acupuncture. The terms *acupuncture* and *dry needling* have been used interchangeably so we have focused on the efficacy of dry needling or acupuncture in scar treatment.

**Objective:**

The aim of this systematic review was to determine the usefulness of dry needling or local acupuncture for scar treatment. In our search process, we used the terms ‘acupuncture,’ ‘needling,’ or ‘dry needling’ to identify all relevant scientific papers. We have focused on the practical aspects of local management of different scar types with dry needling or acupuncture.

**Search strategy:**

The search strategy included different combinations of the following keywords: ‘scar’, ‘keloid’, ‘dry needling’, ‘needling’, ‘acupuncture’, ‘treatment’, ‘physical therapy’. This systematic review was conducted in accordance with PRISMA guidelines. MEDLINE (PubMed, EBSCOHost and Ovid), EMBASE (Elsevier), and Web of Science databases were searched for relevant publications from inception through October 2023.

**Inclusion criteria:**

The studies that investigated the effectiveness of dry needling or acupuncture for scar treatment were included.

**Data extraction and analysis:**

The main extraction data items were: the needling technique; needle: diameter, length; needling locations; manual needling manipulation; number of sessions; settings; outcomes and results.

**Results:**

As a result of a comprehensive search, 11 manuscripts were included in the systematic review, of which eight were case reports, two were randomized trials and one study concerned case series. Two case reports scored 2–4 out of 8 points on the JBI checklist, five studies scored 5–7, and one study scored 8 points. The methodological quality of the two clinical trials was rated as good or fair on the PEDro scale. The case series study scored 7 of 10 points on the JBI checklist. A meta-analysis was not possible as only two randomized trials, eight case reports, and one case series were eligible for review; also, scar assessment scales and pain severity scales were highly heterogeneous.

**Conclusions:**

The studies differed regarding the delivery of dry needling or local acupuncture for scar treatment. Differences included treatment frequency, duration, number of treatments, selection of needle insertion sites, number of needles used, angle of needle placement, and use of manual needling manipulation.

**Systematic review registration:**

INPLASY no. 202310058.

**Supplementary Information:**

The online version contains supplementary material available at 10.1186/s12906-023-04301-4.

## Introduction

Dry needling (DN) therapy uses a thin filiform needle to penetrate the skin and stimulate underlying myofascial trigger points (MTrPs) and muscular, and connective tissues. Dry needling is defined by Western medicine as a type of acupuncture [1]. Acupuncture has been adopted to modern physiotherapy practice based on anatomy, neuroscience, pathology and evidence-based medicine, and has been integrated into the Western medical model [2]. Western medical model acupuncture is now quite commonly used in the treatment of soft tissue injuries within physiotherapy practice [3]. According to Fan et al.(2017), DN can be considered an equivalent of acupuncture, and traditional acupoints are equivalent with trigger points (dry needling points) [4]. In a literature review with implications for clinical practice guidelines, Dunning et al.(2014) noted the terms *acupuncture* and *dry needling* were used interchangeably, and stated that dry needling requires the insertion of thin monofilament needles, as used in the practice of acupuncture [5]. According to Guan-Yuan JIN et al. (2016), dry needling is a type of “contemporary acupuncture because the needles and needling techniques used in dry needling and acupuncture are the same [6]”. Also, Zhu and Most (2016) indicated that dry needling practitioners, such as physical therapists who are not acupuncturists, use the same needles [1]. However, the use of dry needling by physical therapists is not based on ancient theories or tenets of traditional Chinese medicine (TCM) which uses techniques such as acupuncture. A commentary by The American Alliance for Professional Acupuncture Safety (AAPAS) states that dry needling is a subset of acupuncture [7]. Dry needling is used to treat muscles, ligaments, tendons, subcutaneous fascia, and scar tissue [5]. Various explanations of dry needling mechanisms and effects have been proposed. Dunning et al. mention biomechanical, chemical and vascular effects of needling into either superficial subcutaneous tissue (non-muscular) or deep (intramuscular tissue) at trigger point and non-trigger point locations [5]. The potential effects include pain relief, wound healing acceleration, and changes in the neuromyofascial system. Superficial dry needling (SDN) involves insertion of the needle into the subcutaneous tissue, but not the muscle, and seems to adequately address scar tissue.

Scar formation results from wound healing processes that occur following physical injury to body tissues. Prolonged and abnormal wound healing may cause the development of hypertrophic scars which can be itchy and painful, resulting in serious functional disabilities and/or cosmetic defects. It has been suggested that local application of needles around the scars effectively facilitates the scar healing process and alleviates pain and other scar-related symptoms [8]. Therefore, there is a need to continue research to verify the efficacy of local management with needling for scar tissues. This systematic review aimed to assess the usefulness of dry needling or local acupuncture for scar treatment. To identify all relevant scientific papers the terms ‘acupuncture,’ ‘needling,’ or ‘dry needling’ were used in the search process. Practical aspects of dry needling or acupuncture in the local management of different scar types were discussed. In the latter case, we limited our selection to papers describing acupuncture applied locally, mainly in the scar setting.

## Methods

An a priori systematic review protocol was developed and registered at the International Platform of Registered Systematic Review and Meta-analysis Protocols. The registration number is INPLASY 202310058 [9].

This systematic review followed the Preferred Reporting Items for Systematic Reviews and Meta-Analyses (PRISMA) 2020 statement [10].

### Inclusion and exclusion criteria for the review

The research question was defined according to the PICO criteria (Table 1). The eligibility criteria were developed by two reviewers (D.C. and M.N.).

**Table 1:** Inclusion and exclusion criteria defined by the PICO format used in the selection process to identify relevant publications

	Inclusion	Exclusion
Population	Participants of any age: with a scar or keloid or hypertrophic scar	Post-acne scarring, animal and in vitro studies
Intervention	Local management with needling, dry needling or acupuncture, combination of local needling, or dry needling or acupuncture with distal acupuncture	Traditional Chinese Medicine (TCM), wet needling, microneedling, radiofrequency microneedling, trigger point (TrPs) / myofascial trigger point (MTrPs) dry needling beyond scar area, non-therapeutic dry needling, needling with electrical stimulation, electroacupuncture
Comparator	Trials in which the control subjects underwent other conservative treatment (e.g. physical modalities) for scar or did not receive any treatment.	Trials assessing intervention costs, adverse effects only, surgical treatment
Objective*	Changes in pain associated with scar; scar pigmentation, vascularization pattern, shape/thickness, pliability, plasticity, itchiness	Studies on pain/symptoms unrelated to scar / keloid and studies which did not use any scar assessment prior to and after dry needling
Publication type	Full-text research articles in English (randomized controlled trials, clinical trials, case reports, case series, case control studies)	Abstracts, posters, conference proceedings, letters, protocols, reviews (also meta-analyses) and non-clinical trials

*changes assessed using the N*umeric Rating Scale (NRS)*, Visual Analog Scale (VAS) or Likert Scale (e.g., pain, itch); Vancouver Scar Scale (VSS) and POSAS (pigmentation, pliability/plasticity, vascularity); VAS (pruritus/itch); POSAS (thickness and surface area, relief) or other assessment methods

### Information sources

MEDLINE (PubMed, EBSCOHost and Ovid), EMBASE (Elsevier), and Web of Science databases were searched for relevant publications by two lead reviewers (D.C. and M.N.). The databases were searched from their inception until the last entry, between October 5 and 10, 2023. To minimize the risk of relevant sources omission, the strategies to explore Google Scholar were implemented (M.K).

### Search strategy

As mentioned above, the terms *needling*, *dry needling* and *acupuncture* have been used interchangeably. Therefore the search strategy included different combinations of the following keywords: ‘scar’, ‘keloid’, ‘dry needling’, ‘needling’, ‘acupuncture’, ‘treatment’, ‘physical therapy’.

Search strategies for all databases (see Additional file 1) were developed by two reviewers (D.C. and A.O.B.), who gained knowledge in this area through video tutorials and close cooperation with an experienced librarian from the Medical University Library.

### Selection process

All search results were compared; duplicate publications were removed manually by two independent researchers (D.C and M.N.). The ultimate outcome was again compared.

Forms I and II (see Additional file 2) based on inclusion and exclusion criteria were prepared for stages 1 and 2 of the study selection process. Explanation and elaboration documents (see Additional file 3) were also prepared. During the title-abstract stage, two reviewers (D.C. and A.O.B) made independent decisions based on the eligibility criteria presented in Form I to select the retrieved articles. Titles and abstracts lacking sufficient information regarding inclusion criteria specified in Form I were obtained as full texts. Where only the title was available (no abstract), but indicated compliance with the inclusion criteria, the paper was included in stage 2, where the full texts were reviewed. Form II was filled out, and a decision on inclusion of the full text in this systematic review was made. Full texts were independently screened by two reviewers (D.C. and A.O.B.). The reference lists of papers meeting the inclusion criteria were independently searched by two other researchers (M.N. and J.M.) to identify additional relevant studies.

At both stages of the paper selection process, discrepancies between the reviewers regarding eligibility were discussed until a consensus was reached. In cases of uncertainty, an additional reviewer (M.K.) was consulted to make a definitive decision.

### Data collection process

Two reviewers (D.C. and M.N.) collected data from all included studies using a customized data extraction table in Microsoft Excel. They independently copied appropriate extracts from the full texts and pasted them into the table. In the final version of the table, the data extracted by both authors were compared and verified by another researcher (J.M.). In case of disagreement, all three authors debated until a consensus was reached.

### Data items

Data extracted from each study included information related to basic publication characteristics (first author, publication year, country/countries of the research center) and study-specific data (study design, aim of the study, type of scar, sample size, group/s), characteristics of the intervention (e.g. needling technique, needle: diameter, length), its location, manual needling manipulation, number of sessions, setting, outcomes and results. The results were extracted based on the study type.

### Quality assessment. Risk of bias analysis

Assessment of the methodological quality of each study was performed depending on study design. For randomized controlled trials (RCTs), the Physiotherapy Evidence Database (PEDro) score was used to assess the risk of bias and methodological quality of the trials. The Physiotherapy Evidence Database (PEDro) scale consists of 10 questions pertaining to the internal validity and statistical information provided [11]. The total PEDro Scale score is 10 points. Based on the PEDro score, the methodological quality of trials was rated as excellent (PEDro scores 9–10), good (6 to 8 ), fair (4 to 5) or poor (≤ 3) [12]. Two reviewers (D.C. and M.S.) independently assessed the methodological quality of the articles included in this systematic review. In controversial cases, consensus was sought by involving a third researcher (J.M.) [13].

The JBI Critical appraisal tools developed by the Joanna Briggs Institute (JBI) and collaborators, and approved by the JBI Scientific Committee were used for case reports and for case series (https://jbi.global/critical-appraisal-tools). All papers selected for inclusion in this systematic review were subjected to appraisal by two independent reviewers (D.C. and M.S.).

### Data analysis and synthesis

Due to between-study differences regarding certain aspects of treatment and ways to evaluate treatment effects, this systematic review descriptively summarised and analysed the findings from the included studies.

## Results

### Selection of sources of evidence

A total of 924 publication titles and abstracts were identified by searching the electronic databases. After removing duplicates (n = 102), 822 records remained. Google Scholar handsearch yielded another 3 papers. In addition, after scanning the references, one paper was identified as eligible for full-text screening [14].

**Fig. 1:**
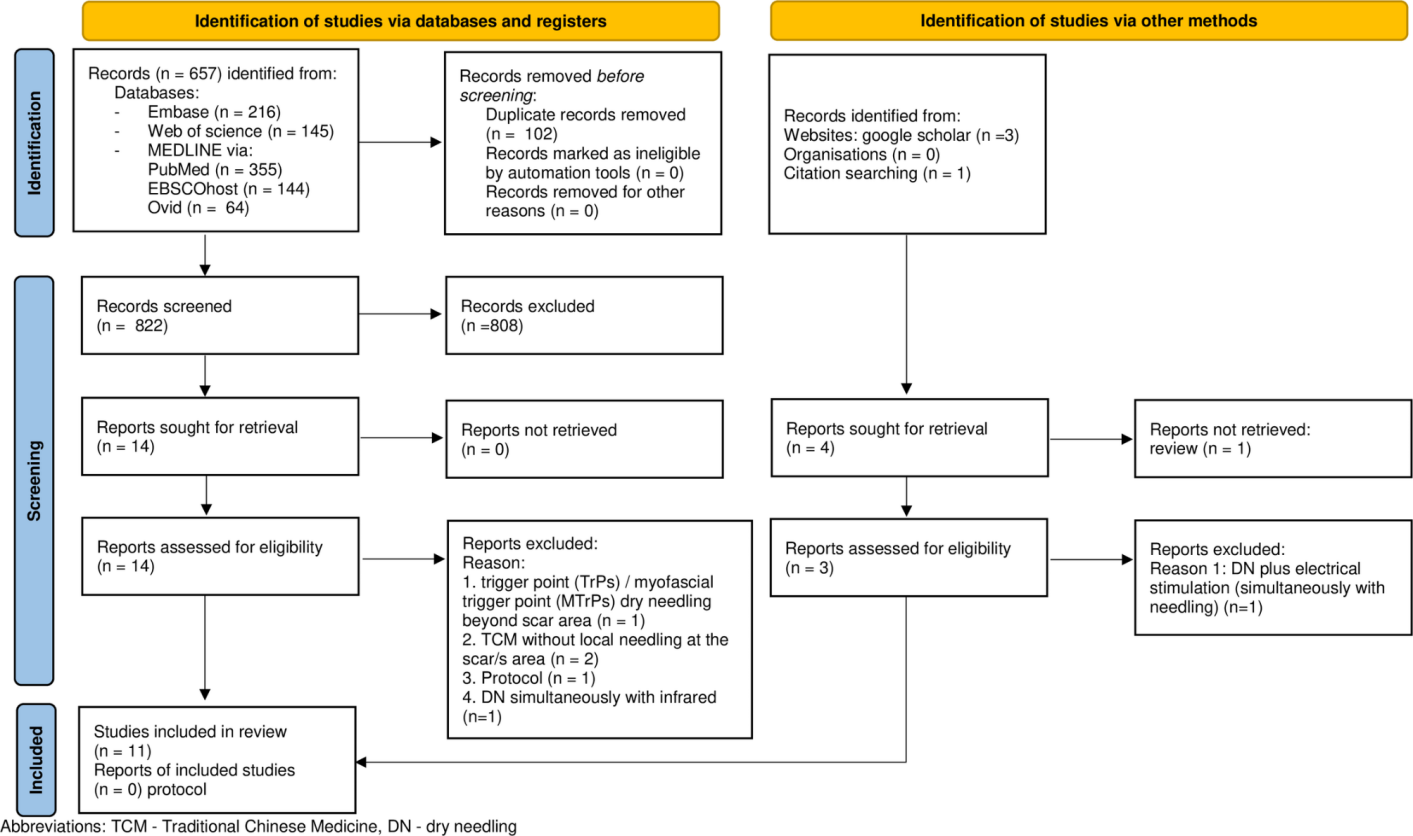
PRISMA diagram flow for the study search and selection

All details of the selection of the sources of evidence, including the reasons for exclusion at the full-text stage, are presented in the PRISMA flow diagram (Fig. 1). The titles of two papers extracted from the electronic databases indicated acupuncture in scar therapy [15, 16]; despite the lack of an abstract, these studies were included in the full-text stage. Following full-text screening (see Additional files 2 and 3), 6 studies were excluded [16–21] and 11 out of 17 publications were considered eligible for this review. Of the 11 publications included in the final review, eight are case reports [14, 15, 22–27], two are randomized clinical trials [28, 29], and one is a case series [30].

### Quality assessment

The JBI Critical appraisal tools were used for case reports included in our review. Two studies scored 2–4 out of a total of 8 points on the JBI checklist [15, 23], five studies scored 5–7 [14, 22–25], and only one study scored 8 points [27] (Table 2). Two low-quality case reports were published as brief reports [15, 23]. Moderate-quality (5–7/8) case reports did not provide information regarding presence or absence of adverse effects of the treatment or unanticipated events were not identified [14, 24, 26]. Only three case studies monitored the risk of side effects of locally performed acupuncture [22, 25, 27]. In three case reports the post-intervention clinical condition was not clearly described, which had an effect on quality assessment (5/8) [23–25]. In some studies, details regarding treatment parameters were not clearly presented [14, 23, 25].

**Table 2:** Eight case reports on the effectiveness of dry needling or acupuncture for scar treatment - assessed using the Joanna Briggs Institute (JBI) appraisal tools

References	1. Were patient’s demographic characteristics clearly described	2. Was the patient’s history clearly described and presented as a timeline	3. Was the current clinical condition of the patient on presentation clearly described	4. Were diagnostic tests or assessment methods and the results clearly described	5. Was the intervention(s) or treatment procedure(s) clearly described	6. Was the post-intervention clinical condition clearly described	7. Were adverse events (harms) or unanticipated events identified and described	8. Does the case report provide takeaway lessons?	*ST
Anderson 2014 [14]	yes	yes	yes	yes	no	yes	no	yes	6
Hunter 2011 [15]	no	no	no	no	yes	yes	no	no	2
Bintoro and Helianthi 2022 [22]	yes	yes	yes	yes	yes	no	yes	no	6
Das and Khan 2019 [23]	yes	yes	yes	yes	no	no	no	no	4
Fang 2014 [24]	yes	no	yes	yes	yes	no	no	yes	5
Huang 2020 [25]	yes	yes	yes	yes	no	yes	yes	yes	7
Tuck 2015 [26]	yes	yes	yes	yes	yes	yes	no	yes	7
Tuckey 2022 [27]	yes	yes	yes	yes	yes	yes	yes	yes	8

*ST - Score Total 0–8 points

Table 3 summarizes the methodological quality of the two randomized clinical trials that were included in our review and rated with the PEDro scale. The methodological quality of these trials was rated as good [28] and fair [29].

**Table 3:** Publications on the effectiveness of dry needling or acupuncture for scar treatment rated with the Physiotherapy Evidence Database (PEDro)

Reference	Eligibility criteria specified	Subjects randomly allocated to groups	Allocation concealed	Groups similar at baseline	Blinding of all subjects	Blinding of all therapists	Blinding assessors	> 85% follow up	Intention-to-treat analysis	Between-group statistical comparison	Point and variability measures	ST*
Kotani et al., 2001 [28]	1	1	1	1	0	0	1	1	1	1	1	**8**
Song et al., 2011 [29]	1	1	0	1	0	0	0	1	1	1	0	**5**

*Eligibility criteria item is not included in the PEDro score calculations

ST- Score Total 10 points. Each item is scored as 1 if present or 0 if absent

The case series study [30] scored 7 of 10 points on the JBI checklist (Table 4).

**Table 4:** One case series on the effectiveness of dry needling or acupuncture for scar treatment - assessed using the Joanna Briggs Institute (JBI) appraisal tools

	Were there clear criteria for inclusion in the case series?	Was the condition measured in a standard, reliable way for all participants included in the case series?	Were valid methods used for identification of the condition for all participants included in the case series?	Did the case series have consecutive inclusion of participants?	Did the case series have complete inclusion of participants?	Was there clear reporting of the demographics of the participants in the study?	Was there clear reporting of clinical information of the participants?	Were the outcomes or follow up results of cases clearly reported?	Was there clear reporting of the presenting site(s)/clinic(s) demographic information?	Was statistical analysis appropriate?	ST
Lubczynska A. et al. 2023 [30]	yes	yes	yes	no	no	yes	yes	yes	no	yes	7

ST - Score Total 0–10 points

### Characteristics of sources of evidence

Two original peer-reviewed research articles published in 2001 [28] and 2011 [29], eight case reports [14–27] published from 2011 to 2022 and one case series [30] published in 2023 were included in the final analysis. The RCTs were from Japan [28] and China [29] (Table 5). The case reports were from the USA [24], UK [26], China [25], Indonesia [22], India [23], Australia and New Zealand [27] and Northern Ireland [14]. The case series was from Poland [30]. In one study the country of origin was not specified [15] (Table 5). The title and/or abstract of one case report (1/8) and one case series included the term *dry needling* [23, 30]; the term *needling* was used in one paper [25] while six used the term *acupuncture* [14, 15, 22, 24, 26, 27]. RCTs titles and/or abstracts included the terms *acupuncture* [29] and *intradermal needling* [28].

**Table 5:** Studies included - dry needling or acupuncture for scar treatment

Reference country	Study objectives	Participant/s characteristics	Type of scar/location of scar/ scar dimension/age of scar	Scar pain/itch rating		Scar assessment - symptoms	
Study design				before needling	before needling	before needling	after needling
Dry needling or acupuncture as a monotherapy in case report studies (n = 5 of 8 case reports included)							
Hunter 2011 [15] (Unspecified) CARE	ACU for keloid scar.	27-year-old female after repair of a Colles’ fracture to the left wrist	Keloid after repair of a Colles’ fracture/ left wrist/6 mos.	Non-specified	Non-specified	Sensitivity	Decrease of sensitivity, pain, thickness
Bintoro and Helianthi 2022 [22] (Indonesia) CARE	To determine the efficacy of a combination of BFA and local point ACU for post- laparotomy scar pain.	69-year-old female after laparotomy, with severe pain located in the area of laparotomy wound	Transverse post- surgical scar between the xiphoid process and the umbilicus/ 30 cm in length and 0.3 cm in width/4yrs.	NRS = 8/11	NRS = 0/11	dark brown and prominent, hardness of tissue along the scar area	changes of tissue color and hardness not reported
Huang et al., 2020 [25] (China) CARE	To present the effects of FNS for subcutaneous adhesions and scar hyperplasia in the neck region.	55-year-old male with impaired neck mobility and difficulty swallowing after tongue lesion resection (lymphadenectomy)	15 cm-long curved post-surgical scar in the neck region, surrounded by numerous scar tissues/8 yrs.	Not measured	Not measured	VSS = total 7 points (M1, V0, H2, P4) Neck ROM: Ex = 38.83 ± 7.25, F = 30.67 ± 7.87; side bend: R = 27.83 ± 3.66, L = 26.00 ± 2.97; rotation: R = 54.83 ± 9.09, L = 53.67 ± 10.82 Cicatricose area with tightly connected scar tissues; other symptoms: affected muscle was tightened, cold, stiff, numb, painful	VSS = total 5 points (M1; V0; H2; P2). Neck ROM: Ex = 41.83 ± 7.33, F = 38.83 ± 3.82; side bend: R = 33.33 ± 2.50, L = 28.33 ± 1.63; rotation: R = 58.33 ± 9.00, L = 62.67 ± 6.54 Reduced soreness, stiffness, cold, tingling, Dissociation of tissue adhesions beneath scars
Das and Khan 2019 [23] (India) CARE	To report the effects of DN on post-scar neuralgia	64-year-old male after hip surgery with pain along the anterolateral aspect of the left thigh	Surgical hip scar and post-scar neuralgia of the left thigh/8yrs.	Non-specified	Non-specified	Non-specified	Non-specified
Fang 2014 [24] (USA) CARE	To report the ACU effects on pain associated with scar tissue.	48-year-old female with stabbing pain in the scar area for 3 mos., sometimes alleviated by ice, and provoked by touch and pressure. Other symptoms: hot flashes, night sweats, dry eyes and photosensitivity; a pale purple tongue body with scallops; menopause at the age of 43 years	Surgical scar/upper right thigh/3 inches long and 1/4 inch wide/1 year.;	Likert Scale 7/10	Likert Scale 1–2/10	tough and hard, red color, very sensitive to touch	no change in toughness and hardness, probably less red in color
Combination of dry needling or acupuncture with other therapeutic modalities in case report studies (n = 3 of 8 case reports included)							
Anderson 2014 [14] (Northern Ireland) CARE	To report the effects of local ACU on the degree of scarring and the ROM of fifth finger after surgical release of Dupuytren’s contracture.	26-year-old male who presented 5 weeks after surgical release of Dupuytren’s contracture of the little finger of his right hand	Keloid scarring over the fifth finger/ 5 weeks	Not measured	Not measured	Fixed F = 25^o^ at the PIPJ, thickness of tissue over the palmar aspect was palpable, altered sensation and numbness in the fifth finger; weakness of the hand extensors; reduced functional ability	Fixed F = 5 ^o^ at the PIPJ, the scar much softer and flatter, improvement in numbness and color in the fifth finger; changes in weakness of hand extensors and functional ability not reported
Tuck 2015 [26] (UK) CARE	To determine the effect of ACU on degenerative lower back pain and neuropathic scar pain.	54-year-old female with scar after metastatic breast cancer and degenerative lower back pain	Post-surgical scar on the chest (right mastectomy and axillary clearance)/4 mos.	scar pain VAS = 3–4/10	no improvement of scar pain VAS = 3–4/10 after superficial needling	Non-specified	Non-specified
Tuckey et al., 2022 [27] (Australia, New Zealand) CARE	To assess the effects of localized ACU for symptomatic scars in a patient with healed burn injury.	71-year-old caucasian male with hypertrophic scar, painful and itchy after burn injury and subsequent skin grafting	Burn scar/skin graft of left lateral thorax /3 mos. and minor burns to the fingers on his left hand	NRS = 7/10 NRS itch = 5/10	NRS = 4.5/10 Follow up NRS = 6/10 NRS itch = 4/10 Follow up NRS itch = 5/10	POSAS = 57/70 (81%) SF-36 - Summary scores (%) PCS – 29, MCS-46	POSAS = 27/70 (38%), Follow up POSAS 33/70 (47%), SF-36 - patient declined to complete the questionnaire Follow up SF-36-unable to complete over the phone
Combination of dry needling or acupuncture with other therapeutic modalities in Randomized Controlled Trials (n = 2)							
Kotani et al., 2001 [28] (Japan) RCT	To determine if insertion of intradermal needles into painful points around scar tissue reduces scar pain.	n = 70 (30 M/40F): Tr group n = 23, 47yr ± 16; Sham tr group = 23,46yr ± 14; C group n = 24, 46yr ± 13. Abdominal scar pain in and around the scar, after acute inflammation, detectable painful points, no satisfactory pain relief with conventional treatments	Surgical scar of abdomen/ at least 12 weeks,	Continuous and lancinating pain assessed using the VAS	Continuous pain - VAS reduction in more than 70% Tr group, lancinating pain - VAS = 0 in more than 40% Tr group, reduction of these parameters < 15% Sham Tr group, no significant pain reduction in C group	area of pain, pressure to initiate painful point pain (pain < = 2.5 kg/cm^2^), daily diclofenac consumption	area of pain: reduction in more than 70% Tr group; less than 15% Sham Tr group pain threshold pressure: increase in Tr group, decrease in Sham Tr group < 15%; daily diclofenac consumption: 70% decrease in Tr group cases, < 15% - Sham Tr group cases, 0% - C group
Song et al., 2011 [29] (China) RCT	To observe the clinical effects of ACU treatment for hypertrophic scar.	n = 80 (44 M/36F), 8–52 yrs. (mean 26 yrs.) with hypertrophic scars, unsmooth surface, congested red color and hard texture, with pain, burning painful, itching sensation or tight sensation	HS different degrees/42 cases-post-operative (chest and abdomen), 23 cases of post-injury scars (four limbs), 6 cases of face scars/ 3 mos. – 4 yrs.(mean 0.7 year.)	Itch assessment as the part of integral criteria	Itch assessment as the part of integral criteria of therapeutic effects	Criteria of therapeutic effects: **cure, effect, failure**. Researchers’ own 3-point scale: 0–3 points for color, itching, hardness. Total scores: 56 of 80 scar sites were severe (9 points), 23 sites were moderate (6–9 points) and 15 sites were mild (1–5 points)	Criteria of therapeutic effects: **cure**: n = 31 Tr group, n = 23 C group; **effect**: n = 15 Tr group, n = 12 C group; **failure**: n = 3 Tr group, n = 10 C group; total effective rate: 93.9% Tr group, 77.8% C group statistically significant difference between the two groups (*P* < 0.01)
Combination of dry needling or acupuncture with other therapeutic modalities in case series study (n = 1)							
Lubczyńska et al. 2023 [30] (Poland) Case series	To assess effectiveness of the scar manual therapy combined with complementary methods on the postoperative scars	n = 11 (F) 32.9 year ± 5.2	Postoperative scar/elbow (n = 1), abdominal (n = 3), and CS (n = 7)/5 mos. (± 2.9)/One person was excluded from the study due to initiation of the other treatment.	PSAS Patient scale NRS = 5 (range 0–10)	PSAS Patient scale NRS ~ 1,5 (range 0–10)	Skin hydration mean = 37.8 ± 7.7 (Corneometer CM 825) TEWL; Tewameter TM Hex) (g/m^2^/h) = 13 ± 4 Stretchability (mean = 0.003 mm ± 0.0003 Erythema level mean = 352.1 ± 103.1 Melanin in the scar tissue POSAS pain, pruritus, color, stiffness, regularity and vascularization, and elasticity.	Skin hydration mean = 48.6 ± 1.2 TEWL (g/m^2^/h) mean = 9.7 ± 2.4 Stretchability (0.05 mm ± 0.01 Erythema level mean = 249.9 ± 89.8 Melanin = no change POSAS = significant changes

Abbreviations: CARE - case reports, ACU - acupuncture, NRS - numeric rating scale; VAS - visual analog scale; VSS - Vancouver Scar Scale, ROM - range of movement: Ex - extension; F - flexion, side bend R- right; L-left, R - rotation R-right; L-left; FNS - Fu’s subcutaneous needling; BFA - Battlefield Acupuncture, M - male, F- female, TCM - Traditional Chinese Medicine, DN - dry needling, RCT- Randomized clinical trial, Tr - treatment group, C group - control group, Sham Tr - Sham treatment group, TBSA - total body surface area, PIPJ - the proximal interphalangeal joint, HS - Hypertrophic scar, POSAS - The Patient and Observer Scar Assessment Scale, SF − 36 - questionnaire Quality of life; MCS - mental component summary, PCS - physical component summary, CS- cesarean section, TEWL - transepidermal water loss

Postsurgical scars were investigated by Fang [24], Tuck [26], Huang et al. [25], Bintoro and Helianthi [22], Das and Khan [23], Kotani et al. [28], and Lubczyńska et al. [30]. Song [29] treated hypertrophic post-operative and post-injury scars; Tuckey et al. [27] discussed acupuncture for burn scars, Hunter [15] and Anderson [14] for keloids (Table 5).

One of the RCTs included 70 individuals [28]; the other RCT comprised 80 subjects [29] (Table 5). The case series included 11 individuals but one participant was excluded from the study due to initiation of the other treatment [30]. The authors of one case report mentioned there was no blinding or sham acupuncture [26]. The remaining case studies provided no information regarding patient or practitioner blinding. RCT studies were randomized [28, 29]. Kotani et al. mentioned that the assessor had been blinded [28]. Only one study provided information on the therapist’s experience in acupuncture [24].

### Characteristics of the intervention

Two studies used dry needling [23, 30] and six studies used local acupuncture as the primary scar treatment [14, 15, 25, 27–29]. Two case reports presented acupuncture to traditional points, combined with acupuncture to local points around the scar [22, 24].

In six studies, acupuncture was combined with other physical modalities including ultrasound [14, 29] and massage [27] or pharmacotherapy [26, 28]. In case series dry needling was used together with manual scar manipulation, massage, cupping, and taping [30] (Table 6).

**Table 6:** Studies included - dry needling or acupuncture for scar treatment

Reference Country	Needling type	Other modalities during therapeutic session	Duration of a single needling session/ treatment frequency	Time of treatment/ number of sessions/ follow-up	Needles: diameter/ length/ manufacturer or material	Needle insertion depth/angle	Scar needling: location or technique/ manual needling manipulation	Study conclusion
Dry needling or acupuncture as a monotherapy in case report studies (n = 5 of 8 case reports included)								
Hunter 2011 [15] (Unspecified)	local ACU	Not applicable	Non-specified	4 mos./8 sessions/6 mos.	0.20 mm/15 mm (Seirin, without guide tube)	Depth not specified/angle non-specified	10 needles inserted locally around the scar 1 cm apart/ „circling the dragon”/ without manipulating	The patient was aware of improvement straightaway; after eight further treatments over four months the scar was flatter and much less sensitive. After six months, the patient was free of pain or other symptoms.
Bintoro and Helianthi 2022 [22] (Indonesia)	local ACU and TCM	Not applicable	30 min	8 wks./ 12 sessions/no follow-up	0.15 mm/15 mm (Huanqiu)	Superficial needling 1-2 mm depth/ angle not specified	a. Inserted locally alongside the scar at points at local areas that had a positive VAS response b. Manual BFA, points on the ear: Cingulate Gyrus, Thalamus, Point Zero, Shen Men, and Omega 2./ without manipulating	A combination of BFA with local-point acupuncture was effective in reducing severe pain caused by a post-laparotomy scar; pain-free, no severe side-effects.
Huang et al., 2020 [25] (China)	Fu’s subcutaneous needling	Not applicable	2 min, 2–3/week	1mo./non-specified/no follow-up	A needle for FSN (Nanjing Paifu Medical Technology Co., Ltd.; Batch No. 20,152,270,832	Subcutaneous depth not specified/at the smallest possible angle into the connective tissues beneath the scars	a. The needle tip 2 cm from the scar, needling direction parallel to the scar tissues /FSN/swaying movement for 2 min beneath scars, frequency of 100 times per minute b. “tightened muscle (TM)”: bilateral SCM, PM, TPA, RA, DIA, ES muscles	FSN therapy has obvious effects on the treatment of muscle-related disorders and can effectively release loose subcutaneous connective tissues and dissociate tissue adhesions beneath scars.
Das and Khan 2019 [23] (India)	DN	Not applicable	1 h	Non-specified/8 sessions/ no follow-up	Non-specified	Depth not specified/angle non-specified	Multiple needles inserted in and around the scar tissue, along pain map of the patient/ without manipulating	The patient reported 50% pain relief.
Fang 2014 [24] (USA)	local ACU and distal TCM points, TCM diagnosis	The patient refused herbs.	20 min/ 2 per week at the beginning, after 3rd session 1 per week	5 wks./8 sessions/no follow-up	0.20 mm/40 mm DBC brand needles	1 cun deep/ inserted at an angle of 45^o^	a. 8 needles inserted 1 cun aside from the scar margins / according to „surrounding the dragon” (Wei Ci technique) / evenly rotated forward and backward; b. TCM distal points: bilateral Hegu-LI-4, Taichong-LIV-3, and Zusanli-ST-36	ACU may have a good short-term pain-relieving effect on scar pain but long-term scar-pain-relieving effects are still unclear.
Combination of dry needling or acupuncture with other modalities during a therapeutic session in case report studies (n = 3 of 8 case reports included)								
Anderson 2014 [14] (Northern Ireland)	local ACU	US, stretching exercises and splintage	20 min	3mos./7 times/no follow-up	a. 0.20 mm/40 mm (no 3) b. 0.3 mm/50 mm (no 8) (Seirin J-Type) depending on the resistance of the scar tissue	Depth not specified/ subcutaneously into the skin at a horizontal angle under the scar	6 needles applied more directly to the scar tissue at local points that induce segmental effects /once stimulated for a few minutes	Measurable improvement in the degree of fixed flexion in the PIPJ of the patient’s little finger, improvements in the temperature, sensation and appearance of the digit.
Tuck 2015 [26] (UK)	local ACU and ACU on the BL meridians	oral morphine	30 min	a.1 session/ in scar location / treatment discontinued b. 2 sessions in back location	a. 0.16 mm /30 mm in scar location (Seirin) b. 0.25 mm /30 mm in back location ACU on the BL meridians (Seirin)	a. 0.5 mm/ perpendicular to the skin in scar location b. 2 cm/ perpendicular to the skin (deep insertion) in back location	a. Local insertion close to the scar line, 1–2 inches away from scar / according to „surrounding the dragon” b. 8 needles/ trigger point in locations: BL22 bilaterally, BL23 bilaterally, BL25 bilaterally and BL26.5 bilaterally (halfway between BL26 and BL27)/ without manipulating	a. local ACU treatment for neuropathic pain secondary to local recurrence in mastectomy scar was unsuccessful. Scar pain did not improve either immediately or within 2 weeks following one local session of acupuncture. b. ACU treatment for low back pain was successful, releasing pain from 7/10 (VAS) to 1/10 (VAS) after two sessions.
Tuckey et al., 2022 [27] (Australia, New Zealand)	local ACU	5 min massage of scar and continued previous regimen of treatment: massage, stretching and exercise	15 min/12 sessions during 7 wks.	7wks./12 sessions/10-week follow up	Non-specified	10 mm depth under the skin/ an angle of 45° (so that 20 mm of the needle shaft was inserted)	20 needles inserted locally at 2 cm intervals (some needles also inserted inside the grafted area) / surrounding the scar / the needles were stimulated manually via bi-directional rotation to the moderate deqi (three times over the course of each session).	ACU applied locally around the scar was associated with short-term relief of symptoms and significantly reduced the patient’s subjective outcome (scar thickness, redness and pliability with a small but clinically relevant reduction in scar pain.
Combination of dry needling or acupuncture with other therapeutic modalities in Randomized Controlled Trials (n = 2)								
Kotani et al., 2001 [28] (Japan)	local ACU	Patients were permitted to take diclofenac during needling treatment	24 h, 5 days per week (Mon-Fri)	4 wks. / 20 sessions/ follow-up weeks 4 and 26	0.16 mm/5 mm (Asahi Industry Co.)	Intradermal depth not specified/ horizontally inserted into each marked skin area	10 local insertion points surrounding the scar detected as painful points (trigger points) in treatment group; nonpainful points in sham group /intradermal/ without manipulating	Insertion of intradermal needles into painful points is an effective treatment for abdominal scar pain. More than 70% of participants in the treatment group showed good- to excellent outcomes, i.e., reduction in all pain parameters. Analgesia was minimal in the sham-treatment and control groups. Decrease in the pain threshold pressure correlated with decreases in continuous and lancinating pain.
Song et al., 2011 [29] (China)	Tr group- local ACU C group without ACU	US- 0.50-1.25cm^2^/ 5–10 min, once a day in Tr group; Mebo Scareducer Ointment + US (the same as Tr group) in C group	30 min/ One every day, 10 sessions per treatment course/ 7-day break x 4 treatment courses	4 courses / 40 sessions / no follow-up	0.30 mm/40-60 mm	Depth not specified/ inserted obliquely at an angle of 15°	Inserted along the skin at the border of the scars / surrounding the scars / needles lifted and thrust 2–3 times and retained for 30 min, and manipulated once every 10 min	ACU plus US therapy is more effective in the treatment of hypertrophic scars. The total effective rate was better in the treatment group.
Combination of dry needling or acupuncture with other therapeutic modalities in case series study (n = 1)								
Lubczyńska et al. 2023 [30] (Poland)	DN	manual manipulation and massage every session cupping (4 times per whole treatment) and taping (8 times per whole treatment)	Non- specified	8wks./ two times per the entire protocol; at 9th and 13th session/no follow-up	Non-specified	Non-specified	“surrounding the dragon” / manipulation non-specified	Treatment had a significant positive effect on pain, pigmentation, pliability, pruritus, surface area, and scar stiffness. Improvement of skin parameters (scar elasticity, thickness, regularity, color) was also noticed.

Abbreviations: TCM -Traditional Chinese Medicine; ACU - acupuncture; DN - dry needling; FNS - Fu’s subcutaneous needling; SCM - sternocleidomastoid muscles, PM - pectoralis major muscles, TPA - trapezius muscles, RA - rectus abdominis muscles, DIA - diaphragm muscles, ES - erector spinae muscles, BFA - Battlefield Acupuncture, US – ultrasound, cun - is traditional Chinese unite of lengths = 3,33 cm

### Duration of a single session, treatment frequency, time of treatment, number of sessions, follow-up

Multiple acupuncture needles were left in situ for different time periods, i.e., 15 to 30 min [14, 22, 24, 26, 27, 29], 2 min [25], and up to 24 h [28]. Needle retention time of 24 h is characteristic for intradermal needling technique [31]. In dry needling techniques, needles were left in situ for 1 h [23]. The frequency of needling treatment sessions was 2 per week [24], 2–3 per week [25], and 5 per week [28] or once daily with 10 sessions making up one treatment course [29], and 12 sessions during 7 weeks [27] or two times per the entire protocol; at 9th and 13th session [30]. No information concerning needling frequency was provided in four case reports [14, 15, 22, 23]. The duration of treatment was 1 month [25], 4 to 5 weeks [24, 28], 7 to 8 weeks [22, 27, 29] or 3 to 4 months [14, 15]. The total number of sessions differed considerably, and ranged from one session [26], two sessions [30], 7–8 sessions [14–23], 12 sessions [22, 27], 20 sessions [28], and up to 40 sessions [29]. The total number of sessions was not given in one paper [25]. Long-term follow-up was carried out at 6 months [15], 4 and 26 weeks [28], and 1 and 2 months of treatment completion [27] (Table 6).

### The needle: diameter, length, depth of insertion, angle of insertion

Needling interventions were heterogeneous. Two RCTs and five case reports provided details on needle diameter and length [14, 15, 22, 24, 26] while the remaining case studies did not [23, 25, 27, 30]. Acupuncture needle diameters were 0.15 mm [22], 0.16 mm [28], 0.20 mm [14, 15, 24] and up to 0.30 mm [29] while needle length was 5 mm [28], 15 mm [15, 22], 30 mm [26], and up to 40-60 mm [14, 29]. In two studies using dry needling the needle diameter and length were not specified [23, 30].

Two case reports and one case series did not specify the depth of needle penetration and insertion angles [15, 23, 30]. Huang [25] and Anderson [14] only mentioned needles had been inserted subcutaneously to the connective tissue beneath the scars at the smallest possible angle. One RCT did not specify the depth of needle insertion, but used oblique insertion at an angle of 15 degrees [29]. In another RCT, intradermal needles were inserted horizontally into painful (treatment group) or nonpainful points (sham group) [28]. Two case studies used superficial needling with needle insertion to depths of 1-2 mm [22] or 0.5 mm perpendicular to the skin [26]. In one case study, the depth of needle insertion was 10 mm under the skin at an angle of 45 degrees [27]. In another one, all needles were inserted about 1 cun deep at an angle of 45 degrees [24] (Table 5).

Two case reports specified the intervals at which the acupuncture needles were placed, i.e., 1 cm [15] and 2 cm [27]. In the other studies, the distance between the needles was determined by the needling technique and needle location. Needles were placed around the pain point (surrounding the dragon technique) [24, 26], into pain points [28], along the pain map determined by the patient [23], in the local area along the scar tissue with a positive VAS response [22], and at local points that induce segmental effects with consequent analgetic effects close to the scar tissue [14].

### Scar needling: location, technique, manual needling manipulation

In the studies of Das and Khan [23], Tuckey [27], Kotani [28] and Song [29], needles were placed at scar margins or around the scar. The technique named ‘surrounding the dragon’ was used by Tuck [26], Fang [24], and Lubczyńska et al. [30], and the ‘circling the dragon’ technique by Hunter [15]. Fu’s subcutaneous needling parallel to scar tissues was applied by Huang [25]. In one case study, the needles were inserted alongside the scar [22]. In five studies manual needle manipulation was employed using the swaying movement [25], bi-directional rotation [24, 27], the ”lifting-thrusting” manipulation [29], or minimal stimulation [14] (Table 6).

### Evaluation of the effectiveness of the therapy

Pain was evaluated using the visual analog scale [26], Likert scale [24], and numeric rating scale [22, 27, 30]. The two case reports did not give any details on pain ratings [15, 23]. In one RCT, a 10-cm VAS was used to evaluate continuous and lancinating pain intensity. The area of pain was measured, and the pressure required to identify a painful point was determined; the patients’ diaries of daily diclofenac consumption were reviewed [28] (Table 5). In most of the studies, the intervention resulted in a reduction of pain around the scar. Tuck reports no mastectomy scar pain improvement either immediately or within 2 weeks following one local session of acupuncture [26]. The patient of Fang [24] reported a decrease of pain after 8 sessions of acupuncture; the pain level dropped from 7 to 1–2 on the Likert scale ranging from 0 to 10 (Table 5). No further improvement was observed after the next two sessions and the patient decided to discontinue the therapy (Table 6). In studies on dry needling patients also reported pain relief [23, 30] (Table 6).

Scar characteristics before and after treatment were assessed using the Vancouver Scar Scale (VSS) [25], and the Patient and Observer Scar Assessment Scale (POSAS) [27, 30]. Song et al. [29] used their own scale where 0 meant no pain, no hardness and normal skin while a score of 3 points indicated serious or constant itch, cartilage-like hardness and dark red or fresh red color (Table 5). Tuckey et al. [27] used the Numerical Rating Scale for pain and itch.

## Discussion

Scar healing is a natural process that occurs as the body repairs and replaces damaged skin tissue. Initially, scars appear red, raised, and sensitive to touch. Over time, the scar tissue gradually remodels and matures, becoming flatter, lighter in color, and less noticeable. While the exact timeline for scar healing can vary depending on the individual and the nature of the injury, the period during which scars are most susceptible to mechanical interventions like needling and ultrasound typically falls within the first six to 18 months after injury occurrence. During this time, the scar tissue is still in the remodeling phase and can benefit from targeted interventions that promote collagen restructuring and stimulate blood flow to the area [32]. While the human body has a remarkable ability to heal itself, there are cases where scars do not heal naturally. Several factors can negatively affect wound healing such as wound severity, infection, poor wound care, tension on the wound, underlying health conditions (e.g. diabetes or autoimmune disorders) or genetic factors causing predisposition to abnormal scarring [33]. In such cases therapeutic interventions including DN may be indicated to promote the healing process and reduce negative symptoms. DN is reported to improve the elasticity of scar tissue; the technique loosens tissues enabling various layers to slide over one another [34].

In the papers presented, the age of the scar ranged from 5 weeks [14], 3–6 months [15, 26–28, 30], 1 year [24], or 4 [22, 29] to 8 years [23, 25], as shown in Table 4. The authors evaluated the scar based on parameters such as pain intensity changes or tissue color changes. This limits the possibility of drawing conclusions about the usefulness of dry needling or acupuncture depending on the age of the scars.

Dry needling and acupuncture are widely believed to lessen scar-related discomfort. Abd-Elsayed et al. report acupuncture has been used for pain control in various clinical conditions associated with chronic scar-related pain [35]. Our review highlights the scarcity of scientific evidence, and RCTs in particular, indicating possible effectiveness of needling for scar thickness, redness, pliability, or restricted range of motion. A meta-analysis was not possible as only two randomized trials and eight case reports were eligible for the review; also scar assessment scales and pain severity scales were highly heterogeneous. Case report studies are known to have a high risk of bias; some do not provide all data on post-intervention clinical condition and changes in symptoms. Anderson, for example, mentioned five pre-acupuncture scar-related symptoms while therapy outcomes were assessed for two [14]. Bintoro and Helianthi did not provide any details on changes in tissue color or hardness [22]. Fang mentioned that post-acupuncture scar was probably less red in color, which indicates only subjective observations were used for scar color evaluation [24]. Das and Khan’s patient reported a 50% reduction in post-scar neuralgia in the left thigh, but it was not stated how the pain severity had been assessed [23]. Some papers had shortcomings in method description. Seven out of the eight case reports are of moderate to low quality ratings according to JBI critical appraisal tools; only one had a maximum score on JBI Checklist [27]. The analysis of the results of the papers selected for review does not clearly establish the effectiveness of acupuncture and dry needling in scar therapy.

Pain severity was assessed using a validated scale in 4 out of 8 case reports and in one RCT. Three studies used the NRS [22, 27, 30], one the Likert scale [24] (although the description indicates it was, in fact, the NRS – *authors’ note*) while two relied on the VAS scores [26, 28]. Twelve sessions combining Battlefield Acupuncture and Local Point Acupuncture resulted in complete resolution of pain complaints [22]. Other papers evaluating the severity of scar-related pain reported some pain reduction after the therapy; no reasons were given for therapy termination although the symptoms did not subside completely. Considering limited data on long-term follow-up, dry needling and local acupuncture cannot be considered effective in scar pain reduction.

The authors of this systematic review were interested in the impact of needling applied to the immediate scar area. Needle insertion in the scar area was used in all studies. The technique known in Chinese medicine as “surround the dragon” [3] was practiced by Tuck [26], Fang [24], Hunter [15], and Lubczyńska et al. [30]. In other research studies, needling was carried out in the immediate vicinity of the scar, around the scar or along the scar. These needling locations are recommended by acupuncturists [31] and dry needling practitioners [8]. Rozenfeld believes needles should be placed around the entire scar or, in the case of scar section being “active” or painful, around the problematic area [8]. Abbate recommends acupuncturists should palpate around the border of the scar and find two most painful places [31]. Tender points needling causes greater pain relief than applying needles to non-painful areas [36]; the same conclusions were drawn by Kotani et al. [28]. The majority of studies selected for our review identified the site of needle insertion based on pain sensation [14, 22–24, 26, 28],. Due to the small number and moderate methodological quality of the papers shortlisted for our review, we cannot conclude about the site of needle insertion in scar therapy in relation to the most painful areas or points in the scar region.

The evidence of the effectiveness of acupuncture therapy alone is not strong since a number of studies have combined needling with other treatment modalities including ultrasound [14, 29], massage [27] and pharmacotherapy [26, 28] or manual manipulation and massage, cupping, taping [30]. It has been confirmed massage had a positive effect on the thickness of hypertrophic and burn scars [37, 38]. The effectiveness of manual manipulation in reducing scar-related problems has been also demonstrated [39, 40]. There is extensive literature on the effects of ultrasound on tissue healing [41–43]. Watson emphasizes that therapeutic ultrasound can increase tensile strength and improve scar mobility by enhancing the appropriate orientation of newly formed collagen fibres and changing the collagen profile [44]. Considering the above, the results should be interpreted as a combined effect of acupuncture and ultrasound [14, 29], massage [27], manual manipulation [30] or other treatment [26, 28, 30].

### Study limitations

This systematic review comprised papers presenting the outcomes of acupuncture and dry needling interventions for scars. However, we agree with Zhou et al., who concluded that although dry needling and acupuncture share similarities, they may differ in certain aspects [45]. Combining these two procedures in one review can therefore be considered a limitation. The scarcity of case reports and RCTs presenting the impact of local acupuncture, and the lack of RCTs on dry needling prevented us from reviewing these two modalities separately. The findings should therefore be interpreted with caution. Also, future reviews should include high quality multi-center trials with uniform criteria, larger sample sizes, standard treatment protocols and outcome measures.

## Conclusions

This systematic review does not allow explicit conclusions on the effectiveness of dry needling or local acupuncture as a monotherapy for scars. The analyzed studies differed regarding the delivery of dry needling or acupuncture for scar treatment. Differences included treatment frequency, duration, number of treatments, selection of needle insertion sites, number of needles used, angle of needle placement, and use of manual needling stimulation. In nine of the ten studies, the dry needling or needling or acupuncture intervention resulted in a reduction of scar pain or other scar-related symptoms.

Multicentre, blinded, randomized, controlled studies on dry needling or acupuncture need to be performed to analyze their effect on scar formation, scar-related pain, and clinical symptoms.

### Electronic supplementary material

Below is the link to the electronic supplementary material.

Additional file 1: Detailed search strategy

Additional file 2: Publication relevance screening form I

Additional file 3: Explanation and elaboration document

## Data Availability

The datasets used and/or analysed during the current study are available from the corresponding author on reasonable request.

## Abbreviations

ACU: Acupuncture
DN: Dry needling
JBI: Joanna Briggs Institute
NRS: Numeric Rating Scale
TCM: Traditional Chinese Medicine
TrPs: Trigger points
SDN: Superficial dry needling
PEDro: Physiotherapy Evidence Database
POSAS: The Patient and Observer Scar Assessment Scale
RCTs: Randomized Controlled Trials
VAS: Visual Analogue Scale
VSS: Vancouver Scar Scale
