# Supplementary material for: Acupuncture and dry needling for physical therapy of scar: a systematic review
Source: BMC Complement Med Ther. 2024 Jan 2;24:14. doi: 10.1186/s12906-023-04301-4 (PMC10759514; doi:10.1186/s12906-023-04301-4)
Supplement: Supplementary file 1 — Additional file 1: Detailed search strategy [file 12906_2023_4301_MOESM1_ESM.docx]

**Additional file1. Detailed search strategy**

| **PubMed**  ((dry needling*[mh]) OR (dry needling*[tiab]) OR (needling*[tiab]) OR (dry needling[tw]) OR (intradermal[tiab]) OR (intradermal[tw]) OR (needling[tw]) OR [acupuncture*[mh] OR (acupuncture*[tiab])) AND ((scar[tiab]) OR (scar[tw] OR (scar[mh]) OR scars[tw])) AND ((therapy[tw]) OR (physical therapy[tw]) OR (treatment[tiab]))  [mh]- MeSH Term  [tiab]- Words and numbers included in the title, collection title, abstract, and other abstract of a citation. English language abstracts are taken directly from the published article  [tw] - Includes all words and numbers in the title, abstract, other abstract, MeSH terms, MeSH Subheadings, Publication Types, Substance Names, Personal Name as Subject, Corporate Author, Secondary Source, Comment/Correction Notes, and Other Terms.  * at the root of a word to find multiple endings |
| --- |
| **Embase**  No.  Query  Results  #25 #11 AND #23 AND [embase]/lim NOT ([embase]/lim AND [medline]/lim) AND [english]/lim  216  #24 #11 AND #23 422  #23 #19 OR #20 OR #21 OR #22 141,796  #22 #16 OR #17 9,026  #21 #18 AND #19 9,152  #20 #14 OR #15 3,527  #19 #12 OR #13 141,295  #18 'hypertrophic' 82,694  #17 'keloid' 9,026  #16 'keloid'/exp 8,164  #15 'skin scar' 3,527  #14 'skin scar'/exp 3,391  #13 'scar' 123,190  #12 'scar'/exp 104,153  #11 #4 OR #7 OR #10 71,339  #10 #8 OR #9 68,863  #9 'acupuncture' 65,789  #8 'acupuncture'/exp 57,862  #7 #5 OR #6 5,583  #6 'needling' AND [embase]/lim 3,907  #5 'needling' 5,583  #4 #1 OR #2 OR #3 1,376  #3 'dry needling' 1,376  #2 'dry needling'/exp 794  #1 'dry needling'/exp AND [embase]/lim 769  exp – (exact Emtree term, exploded), meaning this search not only looks for the subject term you selected but also many related subjects  **# -** Mandated Wildcard **-** Searching with a mandated wildcard retrieves all possible variations of a word in which the wildcard is present in the specified place.  lim-limits |
| **EBSCOhost**  S10 (AB scar) AND (S9)  S9 (S4 OR S6 OR S7) AND (S2)  S8 S4 OR S6 OR S7  S7 ((MH "Dry Needling") or dry needling or needling or intradermal needling) AND (S2 AND S4 AND S6)  S6 (MH "Dry Needling") or dry needling or needling or intradermal needling  S5 (MH "Dry Needling")  S4 (MH "Acupuncture Therapy") OR ( acupuncture or acupuncture therapy or acupuncture treatment )  S3 (MH "Acupuncture Therapy")  S2 (MH "Cicatrix") OR ( scar or keloid )  S1 (MH "Cicatrix")  AB- Abstract. Performs a keyword search of the abstract summaries.  MH- MeSH Term |
| **Ovid MEDLINE**  1 (dry needling* or acupuncture*).af. 42358  2 scar.af. 52568  3 scar.mp. or Cicatrix/ 65607  4. 1 and 2 and 3 ………………………64  af. - All Searchable Fields  mp. = search as Keyword  * at the root of a word to find multiple endings |
| **Web of Science**  Results for "scar" or "keloid" (All Fields) AND "acupuncture" (All Fields) OR "dry needling" (All Fields) NOT "Trigger point*" or "myofascial trigger point*" (All Fields) NOT microneedling (All Fields) and Letter or Meeting Abstract or Early Access or Review Article (Exclude – Document Types) and Sport Sciences or Medicine General Internal (Exclude – Web of Science Categories) and 1.43 Anesthesiology or 1.253 Nerve Disorders (Exclude – Citation Topics Meso) and German or Korean or Turkish or Spanish (Exclude – Languages) |
| Google scholar (05.10.2023yr.)  (dry needling OR needling OR intradermal) OR acupuncture AND scar AND (therapy OR treatment) |
