# Supplementary material for: Acupuncture and dry needling for physical therapy of scar: a systematic review
Source: BMC Complement Med Ther. 2024 Jan 2;24:14. doi: 10.1186/s12906-023-04301-4 (PMC10759514; doi:10.1186/s12906-023-04301-4)
Supplement: Supplementary file 2 — Additional file 2: Publication relevance screening form I [file 12906_2023_4301_MOESM2_ESM.docx]

**Additional file 2. Publication relevance screening form I**

**Title-abstract level**

1. Please, enter the publication title
2. Does the title or abstract concern a scar/keloid/ hypertrophic scar locally managed withdry needling or needling or acupuncture?

- Yes
- No
- If no abstract is available, but the title seems to indicate that the inclusion criteria are met, please obtain the full text of the publication to make the ultimate decision.

1. Does the study concern human subjects?

- Yes
- No

1. Is the publication type an original research article (i.e., a randomized controlled trial, clinical trial, case report, case series, cross-sectional study)?

- Yes
- No

1. Does the title and/or abstract show that the study concerns scar or keloid or hypertrophic scar management using (choose from)

- Traditional Chinese Medicine
- wet needling
- microneedling
- radiofrequency microneedling,
- trigger point (TrPs) / myofascial trigger point (MTrPs) dry needling beyond scar area
- non-therapeutic dry needling/needling
- needling with electrical stimulation
- electroacupuncture
- Yes
- No

1. Have you answered ‘Yes’ to all three questions: 2, 3 and 4?

- Yes
- No

***Publication relevance screening form II***

**Full-text level**

1. Please, enter the publication title
2. Does the paper concern a scar/keloid/ hypertrophic scar locally managed with needling or dry needling or acupuncture?

- Yes
- No

1. Does the paper contain a description of locally performed needling, dry needling, acupuncture?

- Yes
- No

1. Does the paper concern a scar/keloid/ hypertrophic scar managed with a combination of needling or dry needling or acupuncture with distal acupuncture?

- Yes
- No

1. Does the full text concern scar/keloid/ hypertrophic scar management using (choose from)

- Traditional Chinese Medicine acupuncture
- wet needling
- microneedling
- radiofrequency microneedling
- trigger point (TrPs) / myofascial trigger point (MTrPs) dry needling beyond scar area
- non-therapeutic dry needling/needling
- needling with electrical stimualation
- electroacupuncture
- Yes
- No

1. Have you answered ‘Yes’ to questions 2 and 3?

- Yes
- No

1. Have you answered ‘Yes’ to question 4?

- Yes
- No

1. Have you answered ‘Yes’ to question 5?

- Yes
- No
