# Supplementary material for: Acupuncture and dry needling for physical therapy of scar: a systematic review
Source: BMC Complement Med Ther. 2024 Jan 2;24:14. doi: 10.1186/s12906-023-04301-4 (PMC10759514; doi:10.1186/s12906-023-04301-4)
Supplement: Supplementary file 3 — Additional file 3: Explanation and elaboration document [file 12906_2023_4301_MOESM3_ESM.docx]

Additional file 3. Explanation and elaboration document.

Explanation and elaboration:

Reviewer’s Decision on the title and abstract level (Form I):

- if the reviewer’s answer is “Yes” to question nr 6, the publication will be included for further screening, i.e., full-text screening at phase II
- if the reviewer’s answer is “No” to question nr 6, the publication will be excluded from further screening
- if the reviewer’s answer is “Yes” to question nr 5, the publication will be excluded from further appraisal

Reviewer’s Decision on the full text of publication (Form II):

- if the reviewer’s answer is “Yes” to question nr 6, the publication will be included in the review
- if the reviewer’s answer is “Yes” to question nr 7, the publication will be included in the review
- if the reviewer’s answer is “Yes” to question nr 8, the publication will be excluded from the review

Reviewer’s Decision on extracted data shown in the tables:

- if the data in the tables extracted by two independent reviewers are consistent, the text in the tables will undergo formal editing
- if differences in the extracted data are found between the two independent reviewers, a third reviewer will search the full text of the article and will correct the discrepancies in the tables. Any disagreements will be discussed and the final data will be the result of the consensus between the three reviewers.
